# COVID-19 Pandemic Is Associated with an Adverse Impact on Burnout and Mood Disorder in Healthcare Professionals

**DOI:** 10.3390/ijerph18073654

**Published:** 2021-04-01

**Authors:** Yu-Yin Lin, Yu-An Pan, Yi-Ling Hsieh, Meng-Hsuan Hsieh, Yun-Shiuan Chuang, Hsiu-Yi Hsu, Ya-Hsiu Huang, Chia-En Hsu, Yi-Chen Cheng, Shih-Feng Cho, Chao-Ling Wang

**Affiliations:** 1 Health Management Center, Kaohsiung Medical University Hospital, Kaohsiung Medical University, Kaohsiung 807, Taiwan; minami05192000@gmail.com (Y.-Y.L.); amey1125@gmail.com (Y.-A.P.); hsmonyan@gmail.com (M.-H.H.); 2 Department of Occupational Health and Safety Management, Kaohsiung Medical University Hospital, Kaohsiung Medical University, Kaohsiung 807, Taiwan; 1090071@kmuh.org.tw (Y.-L.H.); beautyhsiu02@gmail.com (Y.-H.H.); 1090585@kmuh.org.tw (C.-E.H.); 1080356@kmuh.org.tw (Y.-C.C.); 3 Department of Family Medicine, Kaohsiung Medical University Hospital, Kaohsiung Medical University, Kaohsiung 807, Taiwan; kinkipag@gmail.com; 4 Department of Pediatrics, Kaohsiung Medical University Hospital, Kaohsiung Medical University, Kaohsiung 807, Taiwan; hsoyi@kmu.edu.tw; 5 Division of Hematology and Oncology, Department of Internal Medicine, Kaohsiung Medical University Hospital, Kaohsiung Medical University, Kaohsiung 807, Taiwan; 6 Faculty of Medicine, College of Medicine, Kaohsiung Medical University, Kaohsiung 807, Taiwan; 7 Department of Occupational and Environmental Medicine, Kaohsiung Medical University Hospital, Kaohsiung Medical University, Kaohsiung 807, Taiwan

**Keywords:** coronavirus disease 2019, burnout, mood disorder, healthcare professionals

## Abstract

The coronavirus disease 2019 (COVID-19) pandemic results in a profound physical and mental burden on healthcare professionals. This study aims to evaluate burnout status and mood disorder of healthcare workers during this period. An online questionnaire was voluntarily answered by eligible adult employees in a COVID-19 specialized medical center. The major analysis included the burnout status and mood disorder. Factors related to more severe mood disorder were also identified. A total of 2029 participants completed the questionnaire. There were 901 (44.4%) and 923 (45.5%) participants with moderate to severe personal and work-related burnout status, respectively. Nurses working in the emergency room (ER), intensive care unit (ICU)/isolation wards, and general wards, as well as those with patient contact, had significantly higher scores for personal burnout, work-related burnout, and mood disorder. This investigation identified 271 participants (13.35%) with moderate to severe mood disorder linked to higher personal/work-related burnout scores and a more advanced burnout status. Univariate analysis revealed that nurses working in the ER and ICU/isolation wards were associated with moderate to severe mood disorder risk factors. Multivariate analysis demonstrated that working in the ER (OR, 2.81; 95% CI, 1.14–6.90) was the only independent risk factor. More rest, perquisites, and an adequate supply of personal protection equipment were the most desired assistance from the hospital. Compared with the non-pandemic period (2019), employees working during the COVID-19 pandemic (2020) have higher burnout scores and percentages of severe burnout. In conclusion, this study suggests that the COVID-19 pandemic has had an adverse impact on healthcare professionals. Adequate measures should be adopted as early as possible to support the healthcare system.

## 1. Introduction

Coronavirus disease 2019 (COVID-19), caused by SARS-CoV-2, is a pneumonia of unknown cause first detected in Wuhan, China, and later reported to the WHO Country Office in China on 31 December 2019 [1,2]. Since then, the COVID-19 pandemic rapidly spread globally in 2020. Compared with the previous pandemics of coronaviruses, including severe acute respiratory syndrome coronavirus and Middle East respiratory syndrome coronavirus, COVID-19, is less fatal but much more contagious, with a significantly higher risk of human-to-human transmission even from asymptomatic carriers [3,4,5]. Most patients with COVID-19 have a mild flu-like syndrome or are even asymptomatic [3]. However, approximately 20% of them need hospitalization because of the progression of dyspnea or low oxygen saturation. Additionally, COVID-19 can induce potentially life-threatening organ inflammation, resulting in multiorgan dysfunction and a high mortality rate in a proportion of hospitalized patients, particularly elderly or frail patients [6,7,8,9]. Furthermore, the COVID-19 pandemic represents a multifaceted threat and has had a tremendous impact on the global economy [10].

At the start of the COVID-19 outbreak, little knowledge was available concerning the origins, clinical presentation, or outcomes of COVID-19. With more clinical information revealed, several strategies, including the prevention of disease spread and development of effective drug therapy or vaccines, were implemented and evaluated robustly [11,12,13]. In Taiwan, the first case of COVID-19 was confirmed on 21 January 2020 [14]. Since then, proactive containment efforts, comprehensive contact tracing, and real-time reverse transcription–polymerase chain reaction (RT-PCR) tests to confirm the diagnosis have been utilized to prevent community transmission in Taiwan [13]. All suspected cases, such as close contacts, were quarantined at home for 14 days. If COVID-19-related symptoms developed during quarantine, RT-PCR was performed for confirmation and subsequent management. After the initial increase in confirmed cases, the number of confirmed COVID-19 cases gradually decreased and remained low compared with other countries that had widespread outbreaks [15,16].

After the SARS outbreak in 2003, the Centers for Disease Control of Taiwan established the Communicable Disease Control Medical Network to respond to further outbreaks of infectious disease [17]. During the pandemic period of COVID-19, healthcare professionals play a key role in preventing community or nosocomial outbreak. Several measurements were made, including strict entrance into quarantine, fever screening, the arrangement of patient flow, the partition of hospital zones, and comprehensive survey of recent travel or contact history to every patient [18].

Several efforts trying to control the spread of COVID-19 are also related to increased workload and working hours in frontline healthcare professionals. In addition to daily routine duties, these healthcare professionals spend extra working time on COVID-19 duties. Recent studies have suggested that healthcare professionals in pandemic areas have a higher risk of depression and anxiety, as well as other mental illnesses [19,20]. Additionally, the incidence of mental illness is significantly higher in frontline health workers than non-frontline health workers [21,22]. Due to information being rapidly circulated via various media, the impact of COVID-19 could be widespread [23,24]. To evaluate the potential impact on mental or physical status in a less-affected pandemic area, we conducted this single-institute investigation to identify healthcare individuals at risk of mental distress and evaluate the burnout status and severity with the goal of providing useful information to develop strategies for improving their mental health status.

## 2. Participants and Methods

### 2.1. Study Design

This was a single-institute, nonintervention study using a questionnaire to investigate the burnout index and mental illness during the COVID-19 pandemic. For this study, an online questionnaire was designed and voluntarily answered by eligible adult employees (age ≥ 20 years) of Kaohsiung Medical University Hospital, a tertiary referral hospital and one of the major COVID-19 specialized medical centers in southern Taiwan. The duration of the online questionnaire was from 17 March to 24 May 2020, a period in which the case numbers in Taiwan were rapidly increasing [25]. The data for analysis included age, sex, professional category, and working space/area of participants. The study was conducted based on the Declaration of Helsinki, and the protocol was approved by the Institutional Review Board of Kaohsiung Medical University Hospital (KMUHIRB-E(I)-20200292).

The main analysis for this study was focused on burnout status and mood disorder during the COVID-19 pandemic. First, evaluation of the burnout status included personal burnout and work-related burnout and was conducted using an occupational burnout inventory developed by the Institute of Labor, Occupational Safety and Health, Ministry of Labor of Taiwan, derived from the Copenhagen Burnout Inventory with compatible reliability and validity [26,27,28]. This evaluation comprised six questions, with five scores in each question representing the degree of personal burnout (0, 25, 50, 75, and 100). The personal burnout score was the average score of six questions. Scores of 0, 1 to 49, 50 to 70, and more than 70 (>70) suggest no, mild, moderate, and severe personal burnout, respectively. To evaluate the work-related burnout status, seven questions had five scores on each question representing the degree of the work-related burnout status (0, 25, 50, 75, and 100). The work-related burnout score was the average score of the seven questions. Scores of 0, 1 to 44, 45 to 60, and more than 60 (>60) suggested no, mild, moderate, and severe work-related burnout, respectively. Additionally, psychological symptoms (mood disorder) were evaluated using a five-item brief symptom rating scale (BSRS-5) [29,30]. The score for each item ranged from 0 to 4 (0, not at all; 1, a little bit; 2, moderately; 3, quite a bit; and 4, extremely). A total score on the BSRS-5 above 14 (≥15), or a score of more than 1 on the additional suicide survey item, suggested a severe mood disorder. Scores between 10 and 14, 6 and 9, and 0 and 5 indicated moderate, mild, and no/minimal mood disorder, respectively.

In the final part of the questionnaire, the desired assistance from the hospital was also investigated. The questions were designed as multiple choice, including several alternatives such as more rest, less loading, or perquisites.

### 2.2. Statistics

The analysis for this study included all eligible data. Descriptive statistics were used to summarize the results. Continuous variables, including the number, mean values, standard deviation, and median, were presented. Independent two-sample *t*-tests and paired *t*-tests were used to investigate continuous variables. Categorical variables, including the number and percentages of subjects in each class were presented. Chi-squared tests (*χ*^2^ test) were used to evaluate the frequencies of each categorical variable. The evaluation of correlation was carried out by Pearson’s correlation analysis. To investigate the relative risk of each parameter, univariate and multivariate logistic regression analyses were performed, and odds ratios (ORs) and 95% confidence intervals (CIs) were also calculated. A *p*-value less than 0.05 indicated statistical significance.

## 3. Results

Among 4146 employees, a total of 2029 employees participated and completed the questionnaire, including 355 male and 1674 female, with an overall response rate of 48.9%. The median age was 40 years (range: 21 to 69). The overall mean personal, work-related, and mood disorder scores were 45.38 ± 19.44, 42.78 ± 17.68, and 4.69 ± 3.78, respectively. Regarding the severity of burnout, 901 (44.4%) and 923 (45.5%) participants had moderate to severe personal or work-related burnout, respectively.

**Figure 1:**
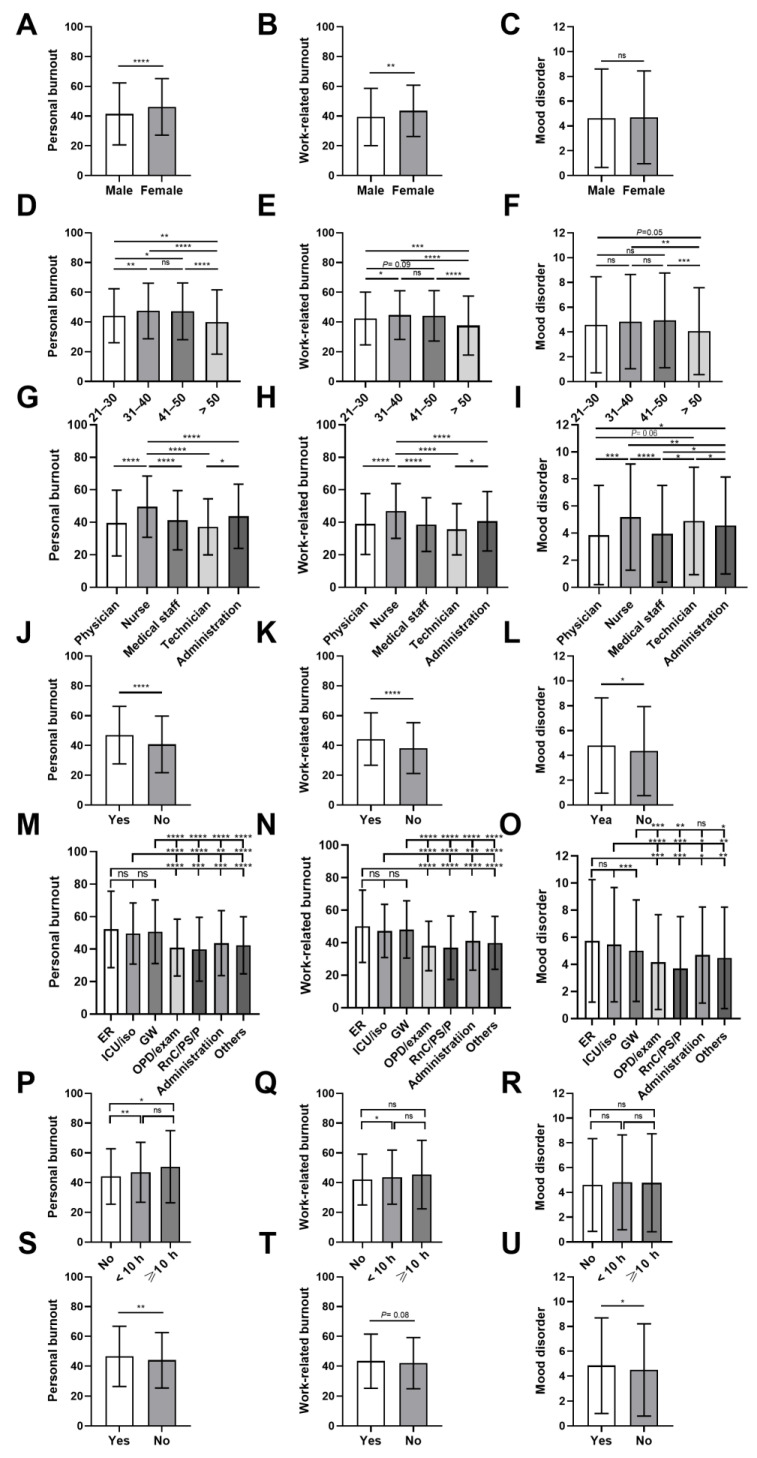
Personal burnout, work-related burnout, and mood disorder scores (mean ± SD) and comparisons between subgroups. The subgroups were stratified by gender (panels **A**–**C**), age (**D**–**F**), professional category (**G**–**I**), patient contact (**J**–**L**), working space/area (**M**–**O**), extra working hours during the COVID-19 pandemic (**P**–**R**), and extra duties for COVID-19 (**S**–**U**) (*, *p* < 0.05; **, *p* < 0.01; ***, *p* < 0.001; ****, *p* < 0.0001; ns, not significant). ER, emergency room; GW, general wards; ICU/iso, intensive care unit or isolation wards; OPD, outpatient department; RnC/PS/P, registration and cashier/patient service/pharmacy.

Comparisons among subgroups were performed. Generally, the female participants had significantly higher scores for personal and work-related burnout than male participants. Additionally, participants aged between 31 and 50 years had higher personal and work-related burnout scores. If the participants were stratified by professional category, nurses had the highest score in personal and work-related burnout, as well as the highest mood disorder score. Furthermore, the participants with direct patient contact had significantly higher personal and work-related burnout scores and higher mood disorder scores. The participants who worked in the emergency room (ER), intensive care unit (ICU), isolation wards, or general wards had higher personal, work-related burnout, and mood disorder scores than other groups (Figure 1). Since the outbreak of COVID-19, several healthcare professionals have been assigned extra duties to prevent community or in-hospital spreading. These participants had higher burnout and mood disorder scores. Additionally, the participants with extra working hours in the COVID-19 pandemic had higher scores for personal burnout (Figure 1). The detailed scores of the subgroups are shown in Supplementary Table S1.

The severity of burnout in each group was analyzed. Female gender, age between 31 to 50 years, nurses, patient contact, and working in the ER, ICU/isolation wards, and general wards were associated with a higher percentage of moderate and severe personal and work-related burnout. The participants with extra working hours (per week) during the COVID-19 pandemic or with extra duties for COVID-19 had a higher percentage of more severe personal burnout. Summaries of the personal and work-related burnout analyses are shown in Supplementary Tables S2 and S3.

Further investigation of the mood disorder status revealed that 271 participants (13.35%) had moderate to severe mood disorder, needing further professional intervention, such as psychological assistance. Compared with the no/minimal or mild mood disorder group, the moderate to severe counterparts had higher percentages of age between 31 and 40 years, were nurses, and worked in the ER, ICU or isolation wards, and general wards. No difference was found between the participants with or without direct patient contact. The basic information of the population with no/minimal, mild, moderate, and severe mood disorder is shown in Table 1.

We next evaluated whether moderate to severe mood disorder was linked to a higher burnout score. First, the group with moderate to severe mood disorder had a significantly higher personal burnout score than their no/minimal or mild counterparts (66.65 ± 19.02 vs. 42.10 ± 17.3, *p* < 0.0001). Additionally, more than eighty percent (*n* = 240, 88.6%) of the participants with moderate to severe mood disorder had a moderate to severe personal burnout. More advanced personal burnout status was also associated with a higher percentage of moderate to severe mood disorder. Regarding the status of work-related burnout, the group with moderate to severe mood disorder also had a significantly higher score than their no/minimal or mild counterparts (62.11 ± 17.08 vs. 39.80 ± 15.80; *p* < 0.0001). A high percentage (*n* = 235, 86.7%) of moderate to severe work-related burnout was also observed in the participants with moderate to severe mood disorder. As with personal burnout, more advanced work-related burnout status was also linked to a higher percentage of moderate to severe mood disorder. (Table 2). The coefficient values of personal burnout scores vs. mood disorder score and work-related burnout scores vs. mood disorder scores were 0.629 and 0.631 (both *p* < 0.0001), respectively, suggesting strong correlations.

The risk factors for moderate to severe mood disorder were evaluated. Univariate analysis revealed nurses working in the ER or ICU/isolation wards were associated with a higher risk of moderate to severe mood disorder. The participants who worked in the general ward showed a trend of a higher risk of moderate to severe mood disorder. When multivariate logistic regression analysis was performed, working in the ER was the only independent factor for moderate to severe mood disorder (OR, 2.81; 95% CI, 1.14–6.90; *p* = 0.024). Additionally, nurses showed a trend for a higher risk of moderate to severe mood disorder (Table 3).

The effects of extra duties for COVID-19 on mood disorder were also evaluated in this study, which revealed that the percentage of moderate to severe mood disorder was similar between the groups (with vs. without duties: 13.5 vs. 13.1, respectively) (Supplementary Table S4). When the participants with extra duties for COVID-19 were further analyzed (total number, *n* = 1062), higher percentages of moderate to severe mood disorder was found in nurses, or those working in the ER, ICU or isolation wards, general wards, and outdoor temporary outpatient departments (OPDs). No significant difference was found in the status of extra working hours between these groups (Supplementary Table S4). The comparisons of the burnout and mood disorder scores among all the subgroups with extra duties for COVID-19 pandemic was also carried out. Female gender had significantly higher burnout (personal and work-related) scores. Nurses had significantly higher burnout (personal and work-related) and mood disorder scores. Patient contact was linked to higher scores in personal and work-related burnout. Extra duties in ER or ICU/isolation wards showed numerically higher scores in personal and work-related burnout, as well as in mood disorder. When these subgroups were stratified by extra working hours per week, only a trend of higher personal burnout scores was observed in the group with more than 10 h of extra working hours. The detailed results and comparisons are shown in Supplementary Figure S1 and Supplementary Table S5.

We further evaluated which type of assistance could substantially assist the study population. Most of the participants needed at least one assessment, and only 79 (3.9%) participants needed no help. More rest (76.7%), perquisites (63.4%), and sufficient personal protective equipment (55.3%) were the three most desired assistances from the hospital. Participants working in the ER, ICU/isolation wards, and general wards had high demands of more rest (78.9%, 88.1%, and 88.9%, respectively) and perquisites (82.2%, 77.7%, and 76.4%, respectively). Among the nurses, 82.1% and 74.6% chose more rest and perquisites as desired assistance, respectively. Compared with the group with no/minimal or mild mood disorder, the moderate to severe counterparts showed higher percentages for more rest, less loading, perquisites for COVID-19 duties, more support from supervisors, psychological consultations, and joining stress relieving groups (Supplementary Table S6).

Finally, to investigate whether the COVID-19 pandemic had a considerable impact on the burnout status, the data collected from the non-COVID-19 pandemic (2019) and COVID-19 pandemic (2020) periods were evaluated. Compared with 2019 (*n* = 3021), the overall personal burnout scores were significantly higher in 2020 (45.38 ± 19.44 vs. 39.04 ± 20.62; *p* < 0.0001). Additionally, higher percentages of participants were identified with severe personal (12.6% vs. 8.5%) and work-related burnout (15.3% vs. 9.1%) during the COVID-19 pandemic (2020). There were 193 participants with moderate to severe mood disorder in 2020 who also completed the burnout status survey in 2019; therefore, we conducted comparisons in this subgroup. The results revealed the personal and work-related burnout scores, as well as the percentages of moderate/severe personal or work-related burnout, were all significantly higher during the COVID-19 pandemic (2020) (Table 4). Among this subgroup, the highest personal and work-related burnout scores were noted in employees working in ER (82.89 ± 22.51 and 78.01 ± 21.96, respectively) in 2020. In addition, increased personal and work-related burnout scores in the pandemic were noted in 149 (77.2%) and 146 (75.6%) employees, respectively. Further analysis showed that the increase in personal burnout scores was remarkable in nurses (*n* = 95, 82.6%), patient contact (*n* = 130, 82.3%), and those working in the ICU/isolation wards (*n* = 25, 86.2%) and general wards (*n* = 49, 81.7%). Increases in work-related burnout scores were observed in employees working in the ER (*n* = 18, 94.7%) and general ward (*n* = 49, 81.7%). Compared with 2019, significantly increased percentages of severe personal burnout in pandemic were observed in nurses (2019: 20%, 2020: 46.1%), administrative staff (2019: 16.2%, 2020: 54.1%), and those working in the ER (2019: 26.3%, 2020: 68.4%) and general wards (2019: 25.0%, 2020: 60%). Similarly, significantly increased proportions of severe work-related burnout were observed in nurses (2019: 20.9%, 2020: 54.8%), administrative staff (2019: 24.3%, 2020: 59.5%), and those working in the ER (2019: 10.5%, 2020: 73.7%), ICU/isolation wards (2019: 17.20%, 2020: 55.2%) and general wards (2019: 23.3%, 2020: 61.7%). The basic characteristics of these 193 participants are shown in Supplementary Table S7. 

## 4. Discussion

Since the beginning of the COVID-19 pandemic, front-line healthcare professionals have been under tremendous work-related or mental stress. In the present study, we investigated the burnout and mood disorder statuses across every professional category and unit/department at a medical center. Our findings suggest that female gender and nurses generally have a higher burnout status during pandemics, a finding likely linked to a higher risk of anxiety and depression [31]. Another previous study also showed more symptoms of depression, anxiety, and poor sleep quality in this subgroup during the COVID-19 pandemic [32]. Regarding the working space, our study also revealed that the ER, ICU, and isolation wards were associated with higher burnout and psychological burdens, similar to another previous study [33]. Additionally, patient contact was also related to significant burnout and mood disorder. The cause may be the higher risk of exposure to infected patients with fever or severe respiratory symptoms.

By multivariate analysis, we identified nurses and the ER as important factors contributing to significant mood disorder, particularly the ER. During the COVID-19 pandemic, frontline nurses in the ER may have the highest risk of infection because of close and frequent contact with patients with fever. Another study also showed that healthcare professionals have a significantly higher risk of SARS-CoV-2 infection than the general population, with most of the infected healthcare workers being nurses [34]. This finding in our investigation is similar to that in another study, which showed a high prevalence rate of depression in ER nurses [35].

Our study showed that almost every participant needed mental or financial assistance from the hospital, or more rest and less work. This finding suggests that the pandemic results in significant stress, leading to an exhausted physical or mental status. Additionally, healthcare professionals are concerned about their own health and the health of their family members. Feeling unsafe and vulnerable in pandemics is linked to poorer mental health [36,37]. Adequate supplies of PPE and timely information on COVID-19 are also very important, because they may provide a perception of safety in these participants [38]. 

In addition to the above findings, the present study also provides solid evidence of the adverse impact of the COVID-19 pandemic on the burnout status in healthcare professionals. Compared with 2019, the personal burnout score and percentage of severe personal and work-related burnout were higher in 2020. No significant difference was found in the overall work-related burnout scores. A possible explanation is the reduced overall behavior in seeking healthcare during the COVID-19 pandemic. Moreover, healthcare professionals may have been used to working in a high-stress environment, making the effect of the COVID-19 pandemic on work-related burnout less significant [39]. However, the personal burnout status could be affected by factors unrelated to work, such as worry or anxiety about the COVID-19 pandemic. Notably, when we analyzed the participants with moderate and severe mood disorder, we found that the personal and burnout scores were significantly increased compared with those in 2019. Moreover, the percentages of the moderate and severe burnout status also clearly increased in 2020. Based on our findings, COVID-19 may exacerbate the burnout status, particularly in participants with potential risk factors for mood disorder.

This study has some strengths and limitations. The analysis for this study contained data from more than two thousand participants, including various professional categories with diverse working characteristics in a less-affected pandemic area. In addition, direct comparison of burnout status between non-pandemic and pandemic periods was also performed, which was lacking in other related studies. With respect to limitations, the response rate was relatively low, which may be due to the short duration of questionnaire investigation. Secondly, this study was a single-institute study; therefore, incorporation of the data from more medical centers may make the study more comprehensive and representative.

In summary, our study revealed that, in this era of rapid information exchange, the global outbreak of COVID-19 still resulted in significant stress on healthcare professionals, even in areas less affected by the pandemic, particularly on frontline healthcare workers. Currently, vaccines for SARS-CoV-2 are under rigorous clinical investigation, and preliminary results have shown high protective ability in early phase clinical trials [40,41,42,43]. The combination of strict infection control and large-scale vaccination programs may further reduce disease spread. However, the development of vaccines has taken substantial time after the outbreak of COVID-19. To support the healthcare system for this long war against the virus, the early deployment of epidemic prevention materials, implementation of strict infection control policies, timely and accurate information, and support programs for employees are critical. In conclusion, our study has evaluated the burnout status, identified a high-risk group of mood disorder and desired assistance, and provides evidence that several supportive measures should be implemented to mitigate stress in the early phase of disease outbreak.

## Figures and Tables

**Table 1:** Characteristics of the study population.

Groups	All (*n* = 2029)	No/Minimal (*n* = 1379)	Mild (*n* = 379)	Moderate (*n* = 227)	Severe (*n* = 44)	*p*-Value
**Gender**						0.66
Male (*n*, %)	355 (17.5)	245 (69.0)	60 (16.9)	40 (11.3)	10 (2.9)	
Female (*n*, %)	1674 (82.5)	1134 (67.7)	319 (19.1)	187 (11.2)	34 (2.0)	
**Age (mean ± SD)**	40.4 ± 10.05	40.7 ± 10.44	40.0 ± 9.14	39.46 ± 9.07	38.27 ± 9.04	
**Age subgroup (years)**						**0.0** **05**
21–30 (*n*, %)	423 (20.8)	294 (69.5)	73 (17.3)	43 (10.1)	13 (3.1)	
31–40 (*n*, %)	621 (30.6)	400 (64.4)	124 (20.0)	86 (13.8)	11 (1.8)	
41–50 (*n*, %)	627 (30.9)	411 (65.6)	132 (21.1)	69 (11.0)	15 (2.4)	
>50 (*n*, %)	358 (17.6)	274 (76.5)	50 (14.0)	29 (8.1)	5 (1.4)	
**Category group**						**<0.00** **01**
Physician (*n*, %)	142 (7.0)	107 (75.4)	22 (15.5)	10 (7.0)	3 (2.1)	
Nurse (*n*, %)	901 (44.4)	561 (62.3)	190 (21.1)	125 (13.9)	25 (2.7)	
Medical staff (*n*, %)	384 (18.9)	295 (76.8)	52 (13.5)	31 (8.1)	6 (1.6)	
Technician (*n*, %)	69 (3.4)	46 (66.7)	12 (17.4)	9 (13.0)	2 (2.9)	
Administration (*n*, %)	533 (26.3)	370 (69.4)	103 (19.3)	52 (9.8)	8 (1.5)	
**Patient contact**						**0.** **081**
Yes (*n*, %)	1491 (73.5)	994 (66.7)	287 (19.2)	172 (11.5)	38 (2.6)	
No (*n*, %)	538 (26.5)	385 (71.6)	92 (17.1)	55 (10.2)	6 (1.1)	
**Working space/area**						**<0.0001**
Emergency room (*n*, %)	90 (4.4)	51 (56.7)	17 (18.9)	20 (22.2)	2 (2.2)	
ICU/isolation wards (*n*, %)	193 (9.5)	116 (60.1)	41 (21.2)	27 (14.0)	9 (4.7)	
General wards (*n*, %)	517 (25.5)	332 (64.2)	105 (20.3)	67 (13.0)	13 (2.5)	
OPD/exam rooms (*n*, %)	441 (21.7)	320 (72.6)	81 (18.4)	32 (7.3)	8 (1.8)	
RnC/PS/P (*n*, %)	98 (4.8)	80 (81.6)	10 (10.2)	5 (5.1)	3 (3.1)	
Administrative area (n, %)	297 (14.6)	201 (67.7)	59 (19.9)	34 (11.4)	3 (1.0)	
Others (n, %)	393 (19.4)	279 (71.0)	66 (16.8)	42 (10.7)	6 (1.5)	
**Extra working hours during COVID-19 pandemic**						**0.** **73**
No (*n*, %)	1201 (59.2)	822 (68.4)	217 (18.1)	140 (11.7)	22 (1.8)	
<10 h (*n*, %)	783 (38.6)	526 (67.2)	155 (19.8)	81 (10.3)	21 (2.7)	
≥10 h (*n*, %)	45 (2.2)	31 (68.9)	7 (15.6)	6 (13.3)	1 (2.2)	

ICU, intensive care unit; OPD, outpatient department; RnC/PS/P, registration and cashier/patient service/pharmacy.

**Table 2:** Summary of the burnout scores of the study population.

Groups	All (*n* = 2029)	No/Minimal (*n* = 1379)	Mild (*n* = 379)	Moderate (*n* = 227)	Severe (*n* = 44)	*p*-Value
**Personal burnout score (mean ± SD)**	45.38 ± 19.44	38.59 ± 16.20	54.85 ± 15.15	64.39 ± 18.11	78.31 ± 19.53	
**Personal burnout severity**						<0.0001
No (*n*, %)	38 (1.9)	38 (100)	0 (0)	0 (0)	0 (0)	
Mild (*n*, %)	1090 (53.7)	952 (87.3)	107 (9.8)	28 (2.6)	3 (0.3)	
Moderate (*n*, %)	645 (31.8)	339 (52.6)	193 (29.9)	106 (16.4)	7 (1.1)	
Severe (*n*, %)	256 (12.6)	50 (19.5)	79 (30.9)	93 (36.3)	34 (13.3)	
**Work-related burnout score (mean ± SD)**	42.78 ± 17.68	36.59 ± 15.03	51.48 ± 12.77	59.94 ± 15.79	73.29 ± 19.23	
**Work-related burnout severity**						<0.0001
No (*n*, %)	20 (1.0)	19 (95.0)	0 (0)	0 (0)	1 (5.0)	
Mild (*n*, %)	1086 (53.5)	941 (86.6)	110 (10.2)	34 (3.1)	1 (0.1)	
Moderate (*n*, %)	612 (30.2)	347 (56.7)	173 (28.2)	86 (14.1)	6 (1.0)	
Severe (*n*, %)	311 (15.3)	72 (23.2)	96 (30.9)	107 (34.4)	36 (11.6)	

**Table 3:** Investigation of the risk of moderate to severe mood disorder.

Variables	Crude OR 95%CI	*p*-Value	Adjusted OR 95%CI	*p*-Value
**Gender group**				
Male	1 (Ref.)			
Female	1.08 (0.77–1.50)	0.657		
**Age subgroup (years)**				
21–30	1 (Ref.)			
31–40	1.21 (0.85–1.73)	0.286		
41–50	1.01 (0.71–1.46)	0.941		
>50	0.69 (0.44–1.08)	0.104		
**Category group**				
Physician	1 (Ref.)		1 (Ref.)	
Nurse	1.98 (1.09–3.59)	0.025	1.70 (0.97–3.11)	0.087
Medical staff	1.06 (0.54–2.05)	0.868	1.07 (0.54–2.12)	0.857
Technician	1.88 (0.80–4.45)	0.150	1.75 (0.72–4.28)	0.220
Administration	1.26 (0.67–2.36)	0.474	1.07 (0.54–2.14)	0.847
**Patient contact**				
No	1 (Ref.)			
Yes	1.282(0.946–1.737)	0.109		
**Working space/area**				
RnC/PS/P	1 (Ref.)		1 (Ref.)	
ER	3.64 (1.53–8.67)	0.004	2.81 (1.14–6.90)	0.024
ICU/isolation wards	2.58 (1.15–5.79)	0.022	1.78 (0.75–4.19)	0.189
General wards	2.51 (0.96–4.41)	0.063	1.45 (0.641–3.30)	0.371
OPD/exam rooms	1.12 (0.51–2.48)	0.776	0.90 (0.40–2.08)	0.809
Administrative area	1.60 (0.72–3.57)	0.249	1.54 (0.66–3.56)	0.315
Others	1.57 (0.72–3.43)	0.262	1.28 (0.57–2.85)	0.554
**Extra duty for COVID-19**				
No	1 (Ref.)			
Yes	1.038 (0.803–1.341)	0.778		

The factors with a *p*-value < 0.1 in the univariate analysis were further analyzed in a multivariate model. ER, emergency room; ICU, intensive care unit; OPD, outpatient department; RnC/PS/P, registration and cashier/patient service/pharmacy.

**Table 4:** Comparison of the personal and work-related burnout between 2019 and 2020 among (1) the overall population in 2019 and 2020 and (2) the participants with moderate/severe mood disorders in 2020.

Overall	2019 (*n* = 3021)	2020 (*n* = 2029)	*p*-Value
Personal burnout score (mean ± SD)	39.04 ± 20.62	45.38 ± 19.44	<0.0001
Personal burnout severity groups			<0.0001
No (*n*, %)	133 (4.4)	38 (1.9)	
Mild (*n*, %)	1916 (63.2)	1090 (53.7)	
Moderate (*n*, %)	716 (23.7)	645 (31.8)	
Severe (*n*, %)	256 (8.5)	256 (12.6)	
Work-related burnout score (mean ± SD)	43.24 ± 12.26	42.78 ± 17.68	0.279
Work-related burnout severity			<0.0001
No (*n*, %)	0	20 (1.0)	
Mild (*n*, %)	1656 (54.8)	1086 (53.5)	
Moderate (*n*, %)	1089 (36.1)	612 (30.2)	
Severe (*n*, %)	276 (9.1)	311 (15.3)	
**Participant with moderate/severe mood disorder in 2020 (also completed 2019 survey)**	**2019**	**2020**	
Personal burnout score (mean ± SD)	48.80 ± 21.49	66.68 ± 19.94	<0.0001
Personal burnout severity groups			<0.0001
No (*n*, %)	4 (2.1)	0	
Mild (*n*, %)	93 (48.2)	24 (12.4)	
Moderate (*n*, %)	59 (30.6)	80 (41.5)	
Severe (*n*, %)	37 (19.1)	89 (46.1)	
Work-related burnout score (mean ± SD)	48.65 ± 13.07	62.10 ± 17.49	<0.0001 ^#^
Work-related burnout severity			<0.0001
No (*n*, %)	0	1 (0.5)	
Mild (*n*, %)	72 (37.3)	28 (14.5)	
Moderate (*n*, %)	81 (42.0)	63 (32.6)	
Severe (n, %)	40 (20.7)	101 (52.3)	

^#^ Analyzed by paired *t*-test.

## Data Availability

The authors confirm that the data supporting the findings of this study are available within the article and its supplementary materials.

## Supplementary Materials

The following are available online at https://www.mdpi.com/article/10.3390/ijerph18073654/s1. Figure S1: Personal burnout, work-related burnout, and mood disorder scores (mean ± SD) and comparisons between subgroups with extra duties for COVID-19 (*n* = 1062); Table S1: Summary of burnout and mood disorder scores (mean ± SD); Table S2: Distribution of personal burnout in all participants; Table S3: Distribution of work-related burnout in all participants; Table S4: Summary of COVID-19-related duties of the study population, classified by the severity of mood disorder; Table S5: Burnout and mood disorder scores in participants with COVID-19-related duties (mean ± SD); Table S6: Summary of the desired assistance in the overall study population. Table S7: Basic characteristics of the participants with moderate/severe mood disorders in 2020 who also completed burnout survey in 2019.

## Abbreviations

COVID-19	coronavirus disease 2019
ER	emergency room
ICU	intensive care unit
OPD	outpatient department

## References

[1] Huang C., Wang Y., Li X., Ren L., Zhao J., Hu Y., Zhang L., Fan G., Xu J., Gu X. (2020). Clinical features of patients infected with 2019 novel coronavirus in Wuhan, China. Lancet.

[2] Zhu N., Zhang D., Wang W., Li X., Yang B., Song J., Zhao X., Huang B., Shi W., Lu R. (2020). A Novel Coronavirus from Patients with Pneumonia in China, 2019. N. Engl. J. Med..

[3] Rothe C., Schunk M., Sothmann P., Bretzel G., Froeschl G., Wallrauch C., Zimmer T., Thiel V., Janke C., Guggemos W. (2020). Transmission of 2019-nCoV Infection from an Asymptomatic Contact in Germany. N. Engl. J. Med..

[4] Helmy Y.A., Fawzy M., Elaswad A., Sobieh A., Kenney S.P., Shehata A.A. (2020). The COVID-19 Pandemic: A Comprehensive Review of Taxonomy, Genetics, Epidemiology, Diagnosis, Treatment, and Control. J. Clin. Med..

[5] Chan J.F., Yuan S., Kok K.H., To K.K., Chu H., Yang J., Xing F., Liu J., Yip C.C., Poon R.W. (2020). A familial cluster of pneumonia associated with the 2019 novel coronavirus indicating person-to-person transmission: A study of a family cluster. Lancet.

[6] Onder G., Rezza G., Brusaferro S. (2020). Case-Fatality Rate and Characteristics of Patients Dying in Relation to COVID-19 in Italy. JAMA.

[7] Guan W.J., Ni Z.Y., Hu Y., Liang W.H., Ou C.Q., He J.X., Liu L., Shan H., Lei C.L., Hui D.S.C. (2020). Clinical Characteristics of Coronavirus Disease 2019 in China. N. Engl. J. Med..

[8] Wang D., Hu B., Hu C., Zhu F., Liu X., Zhang J., Wang B., Xiang H., Cheng Z., Xiong Y. (2020). Clinical Characteristics of 138 Hospitalized Patients With 2019 Novel Coronavirus-Infected Pneumonia in Wuhan, China. JAMA.

[9] Cummings M.J., Baldwin M.R., Abrams D., Jacobson S.D., Meyer B.J., Balough E.M., Aaron J.G., Claassen J., Rabbani L.E., Hastie J. (2020). Epidemiology, clinical course, and outcomes of critically ill adults with COVID-19 in New York City: A prospective cohort study. Lancet.

[10] Pak A., Adegboye O.A., Adekunle A.I., Rahman K.M., McBryde E.S., Eisen D.P. (2020). Economic Consequences of the COVID-19 Outbreak: The Need for Epidemic Preparedness. Front. Public Health.

[11] Lotfi M., Hamblin M.R., Rezaei N. (2020). COVID-19: Transmission, prevention, and potential therapeutic opportunities. Clin. Chim. Acta.

[12] Guan W.J., Chen R.C., Zhong N.S. (2020). Strategies for the prevention and management of coronavirus disease 2019. Eur. Respir. J..

[13] Cheng H.Y., Jian S.W., Liu D.P., Ng T.C., Huang W.T., Lin H.H., Taiwan C.-O.I.T. (2020). Contact Tracing Assessment of COVID-19 Transmission Dynamics in Taiwan and Risk at Different Exposure Periods Before and After Symptom Onset. JAMA Intern. Med..

[14] Cheng S.C., Chang Y.C., Fan Chiang Y.L., Chien Y.C., Cheng M., Yang C.H., Huang C.H., Hsu Y.N. (2020). First case of Coronavirus Disease 2019 (COVID-19) pneumonia in Taiwan. J. Formos. Med. Assoc..

[15] Cheng H.Y., Li S.Y., Yang C.H. (2020). Initial rapid and proactive response for the COVID-19 outbreak—Taiwan’s experience. J. Formos. Med. Assoc..

[16] Wang C.J., Ng C.Y., Brook R.H. (2020). Response to COVID-19 in Taiwan: Big Data Analytics, New Technology, and Proactive Testing. JAMA.

[17] Kao H.Y., Ko H.Y., Guo P., Chen C.H., Chou S.M. (2017). Taiwan’s Experience in Hospital Preparedness and Response for Emerging Infectious Diseases. Health Secur..

[18] Chang Y.T., Lin C.Y., Tsai M.J., Hung C.T., Hsu C.W., Lu P.L., Hou M.F. (2020). Infection control measures of a Taiwanese hospital to confront the COVID-19 pandemic. Kaohsiung J. Med. Sci..

[19] Liang Y., Wu K., Zhou Y., Huang X., Zhou Y., Liu Z. (2020). Mental Health in Frontline Medical Workers during the 2019 Novel Coronavirus Disease Epidemic in China: A Comparison with the General Population. Int. J. Environ. Res. Public Health.

[20] Gold J.A. (2020). Covid-19: Adverse mental health outcomes for healthcare workers. BMJ.

[21] Alshekaili M., Hassan W., Al Said N., Al Sulaimani F., Jayapal S.K., Al-Mawali A., Chan M.F., Mahadevan S., Al-Adawi S. (2020). Factors associated with mental health outcomes across healthcare settings in Oman during COVID-19: Frontline versus non-frontline healthcare workers. BMJ Open.

[22] Cai Q., Feng H., Huang J., Wang M., Wang Q., Lu X., Xie Y., Wang X., Liu Z., Hou B. (2020). The mental health of frontline and non-frontline medical workers during the coronavirus disease 2019 (COVID-19) outbreak in China: A case-control study. J. Affect. Disord..

[23] Gao J., Zheng P., Jia Y., Chen H., Mao Y., Chen S., Wang Y., Fu H., Dai J. (2020). Mental health problems and social media exposure during COVID-19 outbreak. PLoS ONE.

[24] Bao Y., Sun Y., Meng S., Shi J., Lu L. (2020). 2019-nCoV epidemic: Address mental health care to empower society. Lancet.

[25] Chang C.M., Tan T.W., Ho T.C., Chen C.C., Su T.H., Lin C.Y. (2020). COVID-19: Taiwan’s epidemiological characteristics and public and hospital responses. PeerJ.

[26] Yeh W.Y., Cheng Y., Chen M.J., Chiu W.H. (2008). Development and validation of an occupational burnout inventory. Taiwan J. Public Health.

[27] Yeh W.Y., Cheng Y., Chen C.J., Hu P.Y., Kristensen T.S. (2007). Psychometric properties of the Chinese version of Copenhagen burnout inventory among employees in two companies in Taiwan. Int. J. Behav. Med..

[28] Kristensen T.S., Borritz M., Villadsen E., Christensen K.B. (2005). The Copenhagen Burnout Inventory: A new tool for the assessment of burnout. Work Stress.

[29] Lu I.C., Yen Jean M.C., Lei S.M., Cheng H.H., Wang J.D. (2011). BSRS-5 (5-item Brief Symptom Rating Scale) scores affect every aspect of quality of life measured by WHOQOL-BREF in healthy workers. Qual. Life Res..

[30] Lee M.B., Liao S.C., Lee Y.J., Wu C.H., Tseng M.C., Gau S.F., Rau C.L. (2003). Development and verification of validity and reliability of a short screening instrument to identify psychiatric morbidity. J. Formos. Med. Assoc..

[31] Koutsimani P., Montgomery A., Georganta K. (2019). The Relationship Between Burnout, Depression, and Anxiety: A Systematic Review and Meta-Analysis. Front. Psychol..

[32] Lai J., Ma S., Wang Y., Cai Z., Hu J., Wei N., Wu J., Du H., Chen T., Li R. (2020). Factors Associated With Mental Health Outcomes Among Health Care Workers Exposed to Coronavirus Disease 2019. JAMA Netw. Open.

[33] Lu W., Wang H., Lin Y., Li L. (2020). Psychological status of medical workforce during the COVID-19 pandemic: A cross-sectional study. Psychiatry Res..

[34] Barrett E.S., Horton D.B., Roy J., Gennaro M.L., Brooks A., Tischfield J., Greenberg P., Andrews T., Jagpal S., Reilly N. (2020). Prevalence of SARS-CoV-2 infection in previously undiagnosed health care workers in New Jersey, at the onset of the U.S. COVID-19 pandemic. BMC Infect. Dis..

[35] An Y., Yang Y., Wang A., Li Y., Zhang Q., Cheung T., Ungvari G.S., Qin M.Z., An F.R., Xiang Y.T. (2020). Prevalence of depression and its impact on quality of life among frontline nurses in emergency departments during the COVID-19 outbreak. J. Affect. Disord..

[36] Bai Y., Lin C.C., Lin C.Y., Chen J.Y., Chue C.M., Chou P. (2004). Survey of stress reactions among health care workers involved with the SARS outbreak. Psychiatr Serv..

[37] Brooks S.K., Dunn R., Amlot R., Rubin G.J., Greenberg N. (2018). A Systematic, Thematic Review of Social and Occupational Factors Associated With Psychological Outcomes in Healthcare Employees During an Infectious Disease Outbreak. J. Occup. Environ. Med..

[38] Brooks S.K., Webster R.K., Smith L.E., Woodland L., Wessely S., Greenberg N., Rubin G.J. (2020). The psychological impact of quarantine and how to reduce it: Rapid review of the evidence. Lancet.

[39] Tsai Y.C., Liu C.H. (2012). Factors and symptoms associated with work stress and health-promoting lifestyles among hospital staff: A pilot study in Taiwan. BMC Health Serv. Res..

[40] Jackson L.A., Anderson E.J., Rouphael N.G., Roberts P.C., Makhene M., Coler R.N., McCullough M.P., Chappell J.D., Denison M.R., Stevens L.J. (2020). An mRNA Vaccine against SARS-CoV-2—Preliminary Report. N. Engl. J. Med..

[41] Folegatti P.M., Ewer K.J., Aley P.K., Angus B., Becker S., Belij-Rammerstorfer S., Bellamy D., Bibi S., Bittaye M., Clutterbuck E.A. (2020). Safety and immunogenicity of the ChAdOx1 nCoV-19 vaccine against SARS-CoV-2: A preliminary report of a phase 1/2, single-blind, randomised controlled trial. Lancet.

[42] Zhu F.C., Li Y.H., Guan X.H., Hou L.H., Wang W.J., Li J.X., Wu S.P., Wang B.S., Wang Z., Wang L. (2020). Safety, tolerability, and immunogenicity of a recombinant adenovirus type-5 vectored COVID-19 vaccine: A dose-escalation, open-label, non-randomised, first-in-human trial. Lancet.

[43] Baden L.R., El Sahly H.M., Essink B., Kotloff K., Frey S., Novak R., Diemert D., Spector S.A., Rouphael N., Creech C.B. (2021). Efficacy and Safety of the mRNA-1273 SARS-CoV-2 Vaccine. N. Engl. J. Med..

