# Supplementary material for: COVID-19 Pandemic Is Associated with an Adverse Impact on Burnout and Mood Disorder in Healthcare Professionals
Source: Int J Environ Res Public Health. 2021 Apr 1;18(7):3654. doi: 10.3390/ijerph18073654 (PMC8036532; doi:10.3390/ijerph18073654)
Supplement: Supplementary file 1 [file ijerph-18-03654-s001.pdf]

Supplemental data

Supplemental Table S1. Summary of burnout and mood disorder scores (mean ± SD).

	Personal	Work-related	Mood disorder
Gender			
Male	41.47 ± 20.82	39.37 ± 19.26	4.70 ± 3.74
Female	46.21 ± 13.04	43.51 ± 17.26	4.62 ± 3.97
Age (years)			
21–30	44.21 ± 18.15	42.35 ± 17.76	4.59 ± 3.87
31–40	47.45 ± 18.72	44.66 ± 16.39	4.85 ± 3.80
41–50	47.16 ± 19.07	44.15 ± 16.98	4.95 ± 3.82
> 50	40.02 ± 21.62	37.62 ± 19.39	4.07 ± 3.50
Category			
Physician	39.55 ± 20.27	38.96 ± 18.78	3.86 ± 3.66
Nurse	49.62 ± 18.85	46.96 ± 16.85	5.18 ± 3.92
Medical staff	41.33 ± 18.25	38.58 ± 16.54	3.95 ± 3.57
Technician	37.19 ± 17.24	35.71 ± 15.72	4.90 ± 3.97
Administration	43.73 ± 19.76	40.66 ± 18.25	4.56 ± 3.58
Patient contact			
Yes	47.05 ± 19.34	44.39 ± 17.63	4.80 ± 3.85
No	40.77 ± 18.98	38.31 ± 17.08	4.36 ± 3.84
Working space/area			
Emergency room	52.18 ± 23.57	50.15 ± 22.24	5.73 ± 4.52
ICU/isolation wards	49.63 ± 18.86	47.26 ± 16.37	5.48 ± 4.22
General wards	50.72 ± 19.61	48.14 ± 17.62	5.01 ± 3.74
OPD/exam rooms	40.93 ± 17.50	38.03 ± 15.19	4.16 ± 3.50
RnC/PS/P	39.96 ± 19.67	36.92 ± 19.50	3.68 ± 3.84
Administrative area	43.69 ± 19.98	41.08 ± 17.97	4.69 ± 3.54
Others	42.34 ± 17.58	39.91 ± 16.26	4.48 ± 3.74
Extra working hours during COVID-19 pandemic			
No	44.18 ± 18.65	42.06 ± 17.11	4.60 ± 3.74
<10 hours	46.92 ± 20.16	43.73 ± 18.17	4.82 ± 3.83
≥10 hours	50.74 ± 24.25	45.39 ± 23.05	4.78 ± 3.95
Extra duties for COVID-19			
Yes	46.64 ± 20.18	43.42 ± 18.16	4.85 ± 3.84
No	44.00 ± 18.50	42.06 ± 17.14	4.50 ± 3.71

ICU, intensive care unit; OPD, outpatient department; RnC/PS/P, Registration and Cashier/patient service/pharmacy.

Supplemental Table S2. Distribution of personal burnout in all participants.

	No	Mild	Moderate	Severe	*p*-Value
Gender					< 0.0001
Male (n, %)	16 (4.5)	211 (59.4)	88 (24.8)	40 (11.3)	
Female (*n*, %)	22 (1.3)	879 (52.5)	557 (33.3)	216 (12.9)	
Age (years)					< 0.0001
21–30 (*n*, %)	3 (0.7)	262 (61.9)	111 (26.2)	47 (11.1)	
31–40 (*n*, %)	6 (1.0)	313 (50.4)	214 (34.5)	88 (14.1)	
41–50 (*n*, %)	8 (1.3)	311 (49.6)	217 (34.6)	91 (14.5)	
> 50 (*n*, %)	21 (5.8)	201 (57.0)	103 (28.8)	30 (8.4)	
Category					< 0.0001
Physician (*n*, %)	4 (2.8)	97 (68.3)	24 (16.9)	17 (12.0)	
Nurse (*n*, %)	6 (0.6)	415 (46.1)	329 (36.5)	151 (16.8)	
Medical staff (*n*, %)	11 (2.8)	241 (62.8)	98 (25.5)	34 (8.9)	
Technician (*n*, %)	4 (5.8)	43 (62.3)	20 (29.0)	2 (2.9)	
Administration (*n*, %)	13 (2.4)	294 (55.2)	174 (32.6)	52 (9.8)	
Patient contact (*n*, %)					< 0.0001
Yes (*n*, %)	16 (1.1)	779 (52.2)	482 (32.3)	214 (14.4)	
No (*n*, %)	22 (4.1)	311 (57.8)	163 (30.3)	42 (7.8)	
Working space/area					< 0.0001
Emergency room (*n*, %)	1 (1.1)	38 (42.2)	33 (36.7)	18 (20.0)	
ICU/isolation wards (*n*, %)	1 (0.5)	92 (47.7)	70 (36.3)	30 (15.5)	
General wards (*n*, %)	5 (1.0)	234 (45.3)	175 (33.8)	103 (19.9)	
OPD/exam rooms (*n*, %)	10 (2.3)	278 (63.0)	122 (27.7)	31 (7.0)	
RnC/PS/P (*n*, %)	4 (4.0)	57 (58.2)	29 (29.6)	8 (8.2)	
Administrative area (*n*, %)	11 (3.7)	151 (50.8)	104 (35.0)	31 (10.4)	
Others (*n*, %)	6 (1.5)	240 (61.1)	112 (28.5)	31 (10.4)	
Extra working hours during COVID-19 pandemic					0.004
No (*n*, %)	20 (1.6)	684 (57.0)	367 (30.6)	130 (10.8)	
< 10 hours (*n*, %)	17 (2.2)	387 (49.4)	261 (33.3)	118 (15.1)	
≥ 10 hours (*n*, %)	1 (2.2)	19 (42.2)	17 (37.8)	8 (17.8)	
Extra duties for COVID-19					0.003
Yes (*n*, %)	23 (2.1)	535 (50.4)	349 (32.9)	155 (14.6)	
No (*n*, %)	15 (1.6)	555 (57.4)	296 (30.6)	101 (10.4)	

ICU, intensive care unit; OPD, outpatient department; RnC/PS/P, Registration and Cashier/ patient service/pharmacy.

Supplemental Table S3. Distribution of work-related burnout in all participants.

	No	Mild	Moderate	Severe	*p*-Value
Gender					< 0.0001
Male (*n*, %)	10 (2.8)	211 (59.4)	88 (24.8)	46 (13.0)	
Female (*n*, %)	10 (0.6)	875 (52.3)	524 (31.3)	265 (15.8)	
Age (years)					< 0.0001
21–30 (*n*, %)	2 (0.5)	234 (55.3)	113 (26.7)	74 (17.5)	
31–40 (*n*, %)	1 (0.2)	311 (50.1)	212 (34.1)	97 (15.6)	
41–50 (*n*, %)	6 (1.0)	309 (49.3)	212 (33.8)	100 (15.9)	
> 50 (*n*, %)	11 (3.1)	232 (64.8)	75 (20.9)	40 (11.2)	
Category					< 0.0001
Physician (*n*, %)	2 (1.4)	93 (65.5)	30 (21.1)	17 (12.0)	
Nurse (*n*, %)	3 (0.4)	393 (43.6)	314 (34.8)	191 (21.2)	
Medical staff (*n*, %)	2 (0.5)	247 (64.3)	98 (25.5)	37 (9.6)	
Technician (*n*, %)	1 (1.4)	48 (69.6)	18 (26.1)	2 (2.9)	
Administration (*n*, %)	12 (2.3)	305 (57.2)	152 (28.5)	64 (12.0)	
Patient contact (*n*, %)					< 0.0001
Yes (*n*, %)	767 (51.4)		464 (31.1)	260 (17.4)	
No (*n*, %)	339 (63.0)		148 (27.5)	51 (9.5)	
Working space/area					< 0.0001
Emergency room (*n*, %)	0	36 (40.0)	32 (35.6)	22 (24.4)	
ICU/isolation wards (*n*, %)	0	94 (48.7)	56 (29.0)	43 (22.3)	
General wards (*n*, %)	5 (1.0)	200 (38.7)	193 (37.3)	119 (23.0)	
OPD/exam rooms (*n*, %)	5 (1.1)	299 (67.8)	99 (22.4)	38 (8.6)	
RnC/PS/P (*n*, %)	3 (3.1)	60 (61.2)	24 (24.5)	11 (11.2)	
Administrative area (*n*, %)	4 (1.3)	158 (53.2)	96 (32.3)	39 (13.1)	
Others (*n*, %)	3 (0.8)	239 (60.8)	112 (28.5)	39 (9.9)	
Extra working hours during COVID-19 pandemic					0.060
No (*n*, %)	8 (0.7)	670 (55.8)	351 (29.2)	172 (14.3)	
< 10 hours (*n*, %)	11 (1.4)	395 (50.5)	250 (31.9)	127 (16.2)	
≥ 10 hours (*n*, %)	1 (2.2)	21 (46.7)	11 (24.4)	12 (26.7)	
Extra duties for COVID-19					0.131
Yes (*n*, %)	15 (1.4)	546 (51.4)	326 (30.7)	175 (16.5)	
No (*n*, %)	5 (0.5)	540 (55.9)	286 (29.6)	136 (14.0)	

ICU, intensive care unit; OPD, outpatient department; RnC/PS/P, Registration and Cashier/ patient service/pharmacy.

Supplemental Table S4. Summary of COVID-19-related duties of the study population, classified by the severity of mood disorder.

All, *n*= 2029	No/minimal	Mild	Moderate	Severe	*p*-Value
Extra duties for COVID-19					0.242
Yes (*n*, %)	706 (66.5)	212 (20.0)	117 (11.0)	27 (2.5)	
No (*n*, %)	673 (69.6)	167 (17.3)	110 (11.4)	17 (1.7)	
With Extra duties for COVID-19, *n*=1062					
Gender					0.458
Male (*n*, %)	160 (70.2)	39 (17.1)	22 (9.6)	7 (3.1)	
Female (*n*, %)	546 (65.5)	173 (20.7)	95 (11.4)	20 (2.4)	
Age subgroup (year)					0.072
21–30 (*n*, %)	112 (65.5)	34 (19.9)	18 (10.5)	7 (4.1)	
31–40 (*n*, %)	194 (62.8)	72 (23.3)	39 (12.6)	4 (1.3)	
41–50 (*n*, %)	232 (64.7)	73 (20.3)	42 (11.7)	12 (3.3)	
> 50 (*n*, %)	168 (75.3)	33 (14.8)	18 (8.1)	4 (1.8)	
Category					0.040
Physician (*n*, %)	48 (71.6)	13 (19.4)	5 (7.5)	1 (1.5)	
Nurse (*n*, %)	214 (61.3)	73 (20.9)	51 (14.6)	11 (3.2)	
Medical staff (*n*, %)	158 (76.0)	30 (14.4)	15 (7.2)	5 (2.4)	
Technician (*n*, %)	25 (71.4)	7 (20.0)	1 (2.9)	2 (5.7)	
Administration (*n*, %)	261 (64.8)	89 (22.1)	45 (11.2)	8 (2.0)	
Working sites/area (COVID-19)					0.084
Emergency room (*n*, %)	34 (59.6)	12 (21.1)	10 (17.5)	1 (1.8)	
ICU/isolation wards (*n*, %)	46 (56.1)	15 (18.3)	17 (20.7)	4 (4.9)	
General wards (*n*, %)	55 (64.7)	15 (17.6)	11 (12.9)	4 (4.9)	
OPD/exam rooms (*n*, %)	58 (63.7)	22 (24.2)	11 (12.1)	0	
Hospital entrance quarantine	354 (69.4)	103 (20.2)	42 (8.2)	11 (2.2)	
Outdoor temporary OPD	68 (70.8)	12 (12.5)	12 (13.6)	3 (3.1)	
RnC/PS/P (*n*, %)	28 (66.7)	11 (26.2)	3 (7.1)	0	
Administrative area/others (n, %)	63 (63.7)	22 (22.2)	10 (10.1)	4 (4.0)	
Extra working hours during COVID-19 pandemic					0.995
No (*n*, %)	169 (64.8)	54 (20.7)	32 (12.3)	6 (2.3)	
< 10 hours (*n*, %)	507 (67.0)	151 (19.9)	79 (10.4)	20 (2.6)	
≥ 10 hours (*n*, %)	30 (68.2)	7 (15.9)	6 (13.6)	1 (2.3)	

ICU, intensive care unit; OPD, outpatient department; RnC/PS/P, registration and cashier/patient service/pharmacy.

Supplemental Table S5. Burnout and mood disorder scores in participants with COVID-19-related duties (mean ± SD).

Total *n*= 1062	Personal	Work-related	Mood
Gender			
Male	42.58 ± 22.00	39.77 ± 20.09	4.63 ± 3.93
Female	47.74 ± 19.52	44.42 ± 17.47	4.91 ± 3.81
Age (years)			
21–30	45.13 ± 18.73	41.98 ± 18.36	4.75 ± 4.11
31–40	49.11 ± 18.55	45.27 ± 15.59	5.02 ± 3.53
41–50	47.99 ± 20.13	44.90 ± 17.80	5.14 ± 4.06
> 50	42.19 ± 22.67	39.64 ± 21.09	4.22 ± 3.61
Category			
Physician	43.15 ± 22.80	41.31 ± 19.90	4.13 ± 3.71
Nurse	50.69 ± 19.93	46.99 ± 17.44	5.37 ± 4.02
Medical staff	43.21 ± 18.39	40.59 ± 16.45	4.16 ± 3.65
Technician	37.26 ± 20.15	35.92 ± 17.28	4.60 ± 4.23
Administration	46.29 ± 20.17	42.79 ± 18.86	4.89 ± 3.71
Patient contact			
Yes	48.18 ± 20.26	44.87 ± 18.25	4.94 ± 3.96
No	43.43 ± 19.65	40.40 ± 17.60	4.66 ± 3.58
Extra duty sites/area during COVID-19			
Emergency room	52.63 ± 25.83	50.12 ± 24.61	5.68 ± 4.26
ICU/isolation wards	52.74 ± 20.45	49.30 ± 16.63	6.07 ± 4.22
General wards	48.43 ± 20.45	46.55 ± 18.56	5.42 ± 3.91
OPD/exam rooms	43.36 ± 18.71	39.48 ± 15.17	4.81 ± 3.51
Entrance quarantine	46.36 ± 18.84	42.96 ± 16.90	4.50 ± 3.61
Outdoor temporary OPD	46.18 ± 21.65	43.42 ± 20.48	4.76 ± 4.29
RnC/PS/P	43.75 ± 20.18	40.13 ± 18.87	4.55 ± 2.76
Administrative area/others	42.68 ± 21.20	39.32 ± 18.63	4.93 ± 4.36
Extra working hours in COVID-19 pandemic			
No	44.81 ± 19.58	42.01 ± 17.27	4.91 ± 3.86
< 10 hours	47.00 ± 20.91	43.77 ± 18.12	4.83 ± 3.83
≥ 10 hours	51.23 ± 24.31	45.77 ± 23.17	4.82 ± 3.99

ICU, intensive care unit; OPD, outpatient department; RnC/PS/P, Registration and Cashier/ patient service/pharmacy.

Supplemental Table S6. Summary of the desired assistance in the overall study population.

Groups	All (*n*= 2029)	No or mild (*n*= 1758)	Moderate to severe (*n*= 271)	*p*-Value
More rest				< 0.0001
Yes (*n*, %)	1557 (76.7)	1315 (74.8)	242 (89.3)	
No (*n*, %)	472 (23.3)	443 (25.2)	29 (10.7)	
Less loading				< 0.0001
Yes (*n*, %)	1043 (51.4)	865 (49.2)	178 (65.7)	
No (*n*, %)	986 (48.6)	893 (50.8)	93 (34.3)	
Perquisite				0.013
Yes (*n*, %)	1286 (63.4)	1096 (62.3)	190 (70.1)	
No (*n*, %)	743 (36.6)	662 (37.7)	81 (29.9)	
Support from supervisor				0.009
Yes (*n*, %)	625 (30.8)	523 (29.7)	102 (37.6)	
No (*n*, %)	1404 (69.2)	1235 (70.3)	169 (62.4)	
Psychological consultation				< 0.0001
Yes (*n*, %)	342 (16.9)	268 (15.2)	74 (27.3)	
No (*n*, %)	1687 (83.1)	1490 (84.8)	197 (72.7)	
Need stress relieving group				0.009
Yes (*n*, %)	442 (21.8)	366 (20.8)	76 (28.0)	
No (*n*, %)	1587 (78.2)	1392 (79.2)	195 (72.0)	
Adequate COVID-19 information				0.066
Yes (*n*, %)	909 (44.8)	802 (45.6)	107 (39.5)	
No (*n*, %)	1120 (55.2)	956 (54.4)	164 (60.5)	
Sufficient PPE				0.154
Yes (*n*, %)	1122 (55.3)	983 (55.9)	139 (51.3)	
No (*n*, %)	907 (44.7)	775 (44.1)	132 (48.7)	
More in-hospital rest buffer area				
Yes (*n*, %)	616 (30.4)	520 (29.6)	96 (35.4)	0.055
No (*n*, %)	1413 (69.9)	1238 (70.4)	175 (64.6)	

PPE, personal protective equipment.

Supplemental Table S7. Basic characteristics of the participants with moderate/severe mood disorders in 2020 who also completed burnout survey in 2019.

Total *n*= 193	*n* (%)
Gender	
Male (*n*, %)	31 (16.1)
Female (*n*, %)	162 (83.9)
Age (years)	
21–30 (*n*, %)	36 (18.7)
31–40 (*n*, %)	74 (38.3)
41–50 (*n*, %)	63 (32.6)
> 50 (*n*, %)	20 (10.4)
Professional Category	
Physician (*n*, %)	7 (3.6)
Nurse (*n*, %)	115 (59.6)
Medical staff (*n*, %)	28 (14.5)
Technician (*n*, %)	6 (3.1)
Administration (*n*, %)	37 (19.2)
Patient contact (*n*, %)	
Yes (*n*, %)	158 (81.9)
Working space/area	
Emergency room (*n*, %)	19 (9.8)
ICU/isolation wards (*n*, %)	29 (15.0)
General wards (*n*, %)	60 (31.1)
OPD/exam rooms (*n*, %)	28 (14.5)
RnC/PS/P (*n*, %)	5 (2.6)
Administrative area (*n*, %)	52 (26.9)

ICU, intensive care unit; OPD, outpatient department; RnC/PS/P, Registration and Cashier/ patient service/pharmacy.

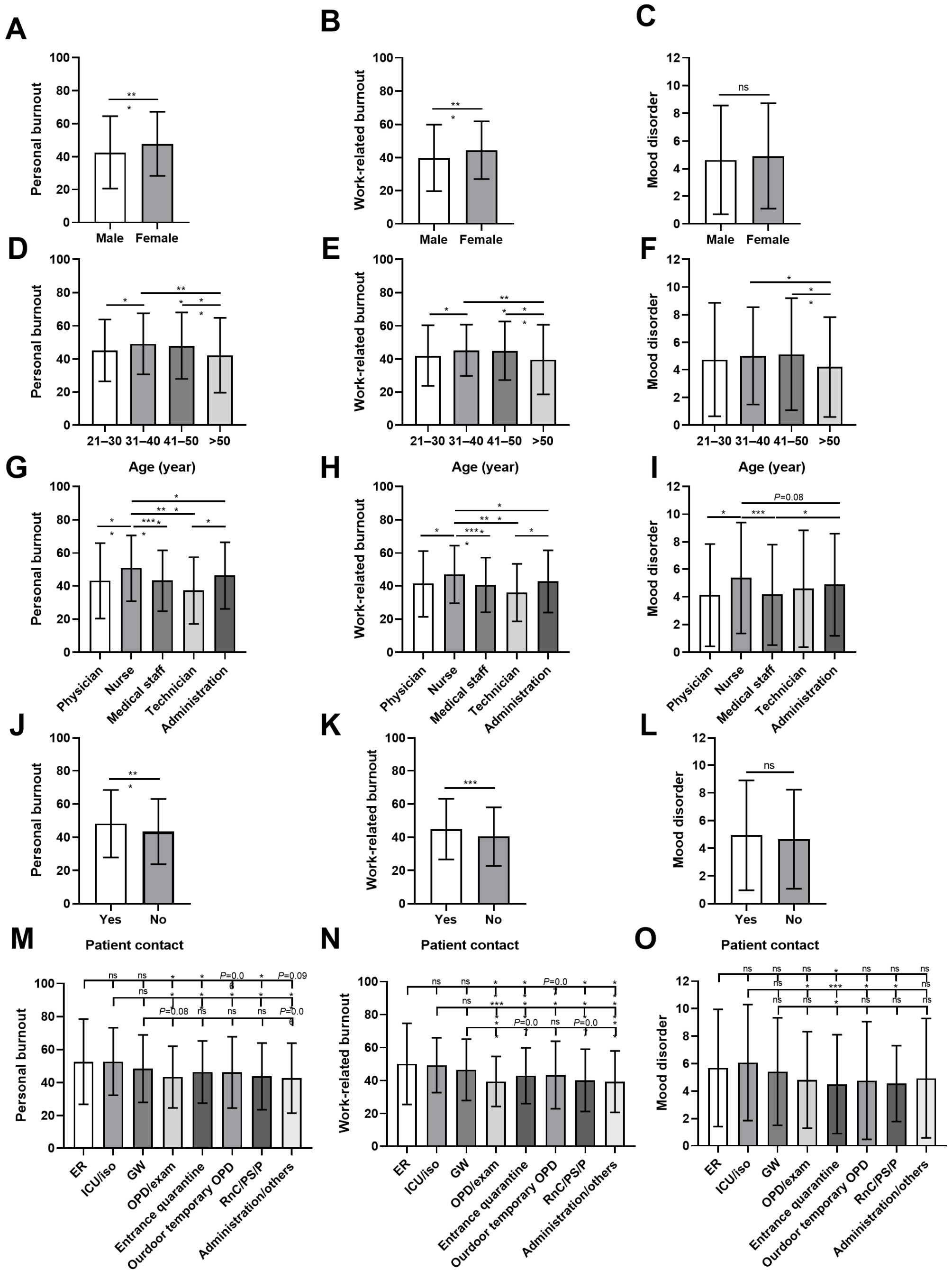

Supplemental Figure S1. Personal burnout, work-related burnout, and mood disorder scores (mean ± SD) and comparisons between subgroups with extra duties for COVID-19 (*n*= 1062). The subgroups were stratified by gender (A, B, C), age (D, E, F), professional category (G, H, I), patient contact (J, K, L), and duty space/area (M, N, O).

(*, $p < 0.05$; **, $p < 0.01$; ***, $p < 0.001$; ****, $p < 0.0001$; ns, not significant). ER, emergency room; GW, general wards; ICU/iso, intensive care unit or isolation wards; OPD, outpatient department; RnC/PS/P, registration and cashier/patient service/pharmacy.
